# Tuning thermal conductivity in molybdenum disulfide by electrochemical intercalation

**DOI:** 10.1038/ncomms13211

**Published:** 2016-10-21

**Authors:** Gaohua Zhu, Jun Liu, Qiye Zheng, Ruigang Zhang, Dongyao Li, Debasish Banerjee, David G. Cahill

**Affiliations:** 1 Materials Research Department, Toyota Research Institute of North America, Ann Arbor, Michigan 48105, USA; 2 Department of Materials Science and Engineering, Frederick Seitz Materials Research Laboratory, University of Illinois at Urbana-Champaign, Urbana, Illinois 61801, USA; 3 Department of Mechanical and Aerospace Engineering, North Carolina State University, Raleigh, North Carolina 27695, USA

## Abstract

Thermal conductivity of two-dimensional (2D) materials is of interest for energy storage, nanoelectronics and optoelectronics. Here, we report that the thermal conductivity of molybdenum disulfide can be modified by electrochemical intercalation. We observe distinct behaviour for thin films with vertically aligned basal planes and natural bulk crystals with basal planes aligned parallel to the surface. The thermal conductivity is measured as a function of the degree of lithiation, using time-domain thermoreflectance. The change of thermal conductivity correlates with the lithiation-induced structural and compositional disorder. We further show that the ratio of the in-plane to through-plane thermal conductivity of bulk crystal is enhanced by the disorder. These results suggest that stacking disorder and mixture of phases is an effective mechanism to modify the anisotropic thermal conductivity of 2D materials.

## Abstract (web summary)

Molybdenum disulfide shows potential for use in nanoelectronics and optoelectronics. Here, the authors use electrochemical intercalation to tune its structural and compositional features, and show that the thermal conductivity can be effectively modified over a considerable range.

Two-dimensional (2D) layer-structured materials consist of atomic layers with strong intra-layer covalent bonding stacked together by weak van der Waals bonds. Transition metal dichalcogenides, an important class of 2D materials, have attracted extensive research interest recently due to their unique electronic and chemical properties^1^. In particular, molybdenum disulfide (MoS_2_) has been extensively studied for potential applications in nanoelectronics, optoelectronics and flexible electronic devices^2^,^3^,^4^,^5^. Although the thermal conductivity of single, few-layer and bulk MoS_2_ has been reported recently, the effects of structural and compositional disorder on the anisotropic thermal conductivity of layered materials, which usually occurs during crystal growth, fabrication and applications (for example, in energy storage, thermoelectrics and nanoelectronics), have not yet been systematically characterized^6^,^7^,^8^.

Guest ions can be intercalated into the van der Waals gaps in MoS_2_. Intercalation causes changes in the electronic structure, and optical and electrical properties^9^. Intercalation can also induce structural and compositional disorder, including variations in layer spacing, interaction strengths between adjacent layers and phase transitions^10^,^11^,^12^. By monitoring the potential during electrochemical intercalation, we can control the amount of intercalated ions. Therefore, intercalation provides an effective way to systematically vary the structural and compositional disorder of many 2D materials, and enables investigations of how disorder affects their thermal conductivity.

To understand how thermal conductivity of highly anisotropic materials can be affected by disorder, here we study the thermal conductivity of both pristine and lithium ion-intercalated bulk and thin-film MoS_2_. In the bulk sample, the MoS_2_ basal planes are oriented parallel to the surface; whereas, in the thin-film sample, the MoS_2_ basal planes are vertically aligned. The thermal conductivity of Li_x_MoS_2_ samples with different degrees (*x*) of electrochemical interaction of lithium ions were measured by time-domain thermoreflectance (TDTR). We show that lithium ion intercalation has drastically different effects on thermal transport in these different forms of MoS_2_ due to the differences in crystalline orientation and initial structural disorder. Our most striking observation is that the thermal anisotropy ratio in bulk Li_*x*_MoS_2_ crystals increases from 52 (*x*=0) to 110 (*x*=0.34) as a result of lithiation-induced stacking disorder and phase transitions. The thermal anisotropy ratio is the ratio of in-plane to through-plane thermal conductivity, an important material parameter in thermal management. The increase in thermal anisotropy with increasing disorder is counter-intuitive: previous studies show that structural disorder typically decreases the thermal anisotropy ratio^13^,^14^. Our analysis suggest that the enhanced thermal anisotropy ratio in Li_*x*_MoS_2_ bulk crystal is likely due to the combination of phonon-focusing effects and pronounced differences in the in-plane and through-plane length scale of the lithiation-induced disorder.

## Results

### Sample preparation

**Figure 1:**
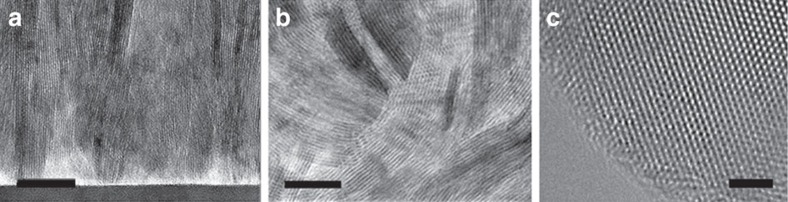
TEM images of MoS_2_ bulk crystals and thin films. (**a**) Cross-sectional TEM image of the MoS_2_ thin film with vertically aligned basal plane. Scale bar, 20 nm. (**b**) Plan-view TEM image of the MoS_2_ thin film. Scale bar, 10 nm. (**c**) Plan-view TEM image of the bulk MoS_2_ crystal. Scale bar, 2 nm.

The MoS_2_ thin-film samples with vertically aligned basal planes were grown by rapid sulfurization of a Mo thin film^15^. Bulk samples of MoS_2_ were obtained by mechanical exfoliation of bulk MoS_2_ crystals (SPI Supplies). The cross-sectional transmission electron microscopy (TEM) image in Fig. 1a reveals that the film thickness is ≈200 nm after chemical-vapour deposition (CVD) growth and the MoS_2_ atomic layers are predominantly aligned perpendicular to the substrate with the edges of the MoS_2_ layers exposed to the surface. A typical plan-view TEM image of the vertically aligned MoS_2_ thin film is shown in Fig. 1b. The thin film is polycrystalline with randomly oriented strip-like or columnar grains. The cross-sectional area of those columnar grains is ∼10 nm wide and several tens of nanometres long. Bulk MoS_2_ samples, with typical thickness of 10–20 μm, were prepared by standard Scotch tape assisted mechanical exfoliation. A plan-view TEM image of the bulk MoS_2_ sample is shown in Fig. 1c, where the high-quality atomic plane of MoS_2_ can be seen.

Further structural analysis of bulk and thin-film MoS_2_ samples before lithiation were carried out by X-ray diffraction and Raman spectroscopy. In X-ray diffraction, bulk samples show a strong (002) peak located at 2*θ*=14.4^o^, and (004), (006) and (008) peaks with decaying magnitudes, indicating that the basal planes of the bulk sample are parallel to the surface. The thin-film samples have two peaks located at 32.8^o^ and 58.5^o^, which are due to the diffraction from (100) and (110) planes of individual columnar grains, consistent with our conclusion from the TEM images that the basal planes are vertically aligned (see details in Fig. 5). In contrast to the bulk sample, (002) peak is not observed in the X-ray diffraction spectrum of the thin-film sample because the normal to the (002) planes is parallel to the substrate surface.

**Figure 2:**
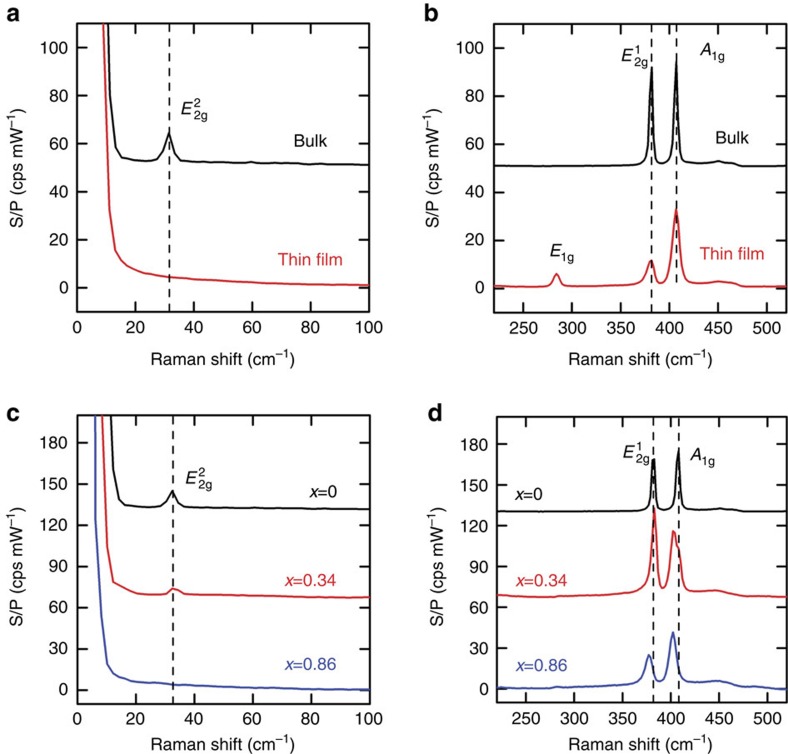
Raman spectra for bulk and thin-film MoS_2_ samples before lithiation. Spectra obtained at (**a**) low frequencies and (**b**) high frequencies. The *y*-axis is the signal intensity normalized by laser power (S/P) in the unit of counts per second per milliwatt (cps mW^−1^). The bulk spectrum is shifted up by 50 cps mW^−1^. Raman spectra for bulk Li_*x*_MoS_2_ samples at different degrees of lithiation (*x*=0, 0.34 and 0.68) at (**c**) low frequencies, (**d**) high frequencies. The *x*=0.34 and *x*=0 spectra are shifted up by 65 and 130 cps mW^−1^, respectively.

Figure 2a,b present the Raman spectra of the bulk and thin-film MoS_2_ samples. To probe the low-frequency interlayer 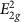 phonon mode, we used three reflective volume Bragg grating filters (BragGrate notch filters) in combination with a single-pass monochromator to access frequency shifts as small as ∼10 cm^−1^. The *E*_1*g*_ mode (286 cm^−1^) is observed only in the thin-film samples while the 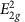 mode (32 cm^−1^) is only present in the bulk samples, as expected. According to the Raman selection rules, the *E*_1*g*_ mode is forbidden in backscattering experiment on the basal plane of bulk MoS_2_ (refs 16, 17). However, when the incident light scatters on the surface of edge-terminated MoS_2_, the corresponding scattering Raman tensor undergoes a rotation transformation, leading to a non-zero differential scattering cross-section and hence the *E*_1*g*_ mode can be observed. The observation of the *E*_1*g*_ mode in thin-film samples therefore indicates that the basal planes of MoS_2_ are vertically aligned, consistent with the TEM and X-ray diffraction data. The absence of the 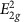 mode (which is not forbidden by selection rules) in thin-film samples is probably due to the randomly oriented columnar grains and stacking disorder in CVD-grown samples. In addition, although both *A*_1*g*_ at 383 cm^−1^ and 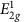 at 408 cm^−1^ modes are present in bulk and thin-film MoS_2_, the peak intensity of the out-of-plane 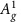 mode is similar to that of the in-plane 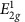 mode in the bulk sample and ∼3 times that of the 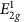 mode in the thin-film sample under the same measurement condition. Such preferred excitation of an out-of-plane mode is also consistent with the vertical-aligned crystal texture of the thin-film sample considering the polarization dependence of the Raman scattering cross-section^15^.

We carried out electrochemical intercalation of lithium ions in both bulk and thin-film MoS_2_ to study how lithiation affects the thermal conductivity differently in MoS_2_ samples with different orientations. Lithium ion intercalation of thin-film MoS_2_ samples was performed through a galvanostatic discharge process in a glass vial inside a glovebox; electrochemical intercalation of bulk MoS_2_ samples as performed using a coin cell battery setup^18^. In both cases, MoS_2_ samples were used as the working electrode, and the lithium foil was used as the counter and reference electrode.

**Figure 3:**
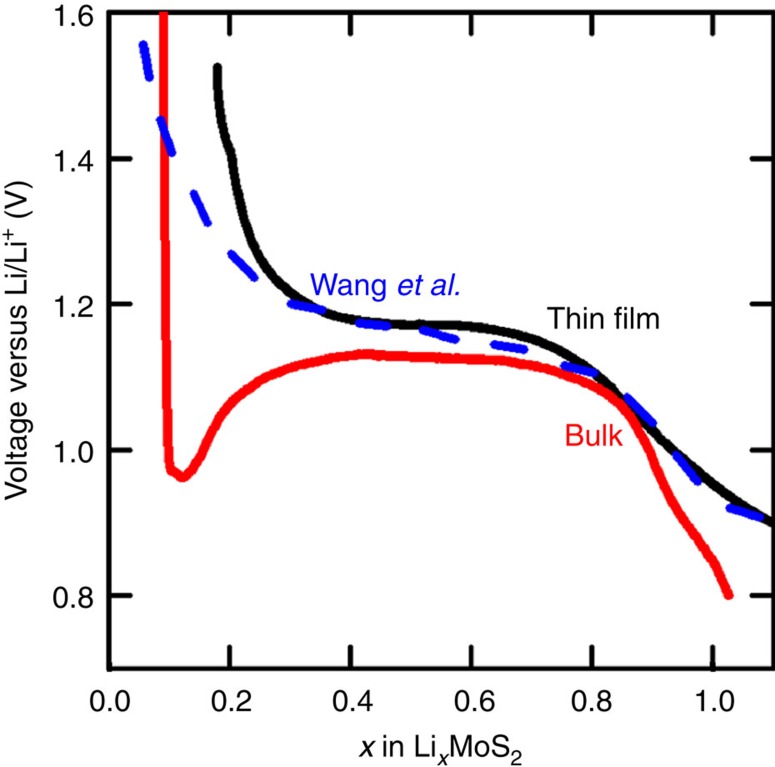
Initial discharge curves for MoS_2_ samples. Thin-film (black) and bulk (red) MoS_2_ samples, compared with data by Wang *et al*.^55^ (dashed line) for thin-film Li_*x*_MoS_2_.

The discharge curves for thin-film and bulk MoS_2_ samples are shown in Fig. 3. On lithium ion intercalation, a well-defined plateau is observed at potentials between 1.1 and 1.2 V, as the host lattice of MoS_2_ undergoes a phase transition from 2H to 1T phase^19^. The voltage dip at the initial stage of the discharge curve for bulk MoS_2_ sample is caused by the mass transport limitation of lithium ions. The voltage gradually recovers when the lithium ion transport is facilitated by defects formed during intercalation. The voltage dip was not observed in the discharge process of thin-film MoS_2_, which can be attributed to the high density of the edge sites in edge-terminated thin-film samples.

We describe the lithium ion intercalation process in the range of 1.1–3.0 V as reaction (1):


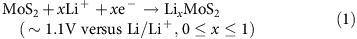


We calculate the average lithium composition from the electronic charge transferred to MoS_2_, based on the theoretical specific charge capacity for full intercalation of 167 mA h g^−1^ (ref. 19). Hence, by controlling the duration of the galvanostatic discharge process as shown in Fig. 3, vertically aligned Li_*x*_MoS_2_ thin-film (*x*=33 at%, 46 at%, 68 at% and 100 at%) and lithiated bulk samples (20 at%, 40 at%, 60at%, 80 at% and 100 at%) with various lithiation content (*x*) were prepared.

Since the charge transfer during electrochemical intercalation can have a contribution from complicated side reactions, the actual degree of lithiation (*x*) for each sample might be different from the calculated values. To determine the actual *x* in bulk MoS_2_ samples, inductively coupled plasma mass spectroscopy measurements were performed on one fully lithiated bulk sample. The mass ratio of Li, Mo and S atoms is calculated by measuring the element masses in the bulk Li_*x*_MoS_2_ sample with metal coating (total mass≈1 mg). The measured *x* is 86 at% suggesting reasonable agreement between calculated and measured lithium contents for the lithiation process. The actual *x* in other bulk Li_*x*_MoS_2_ samples is then scaled by the same factor of 0.86.

### Thermal conductivity

**Figure 4:**
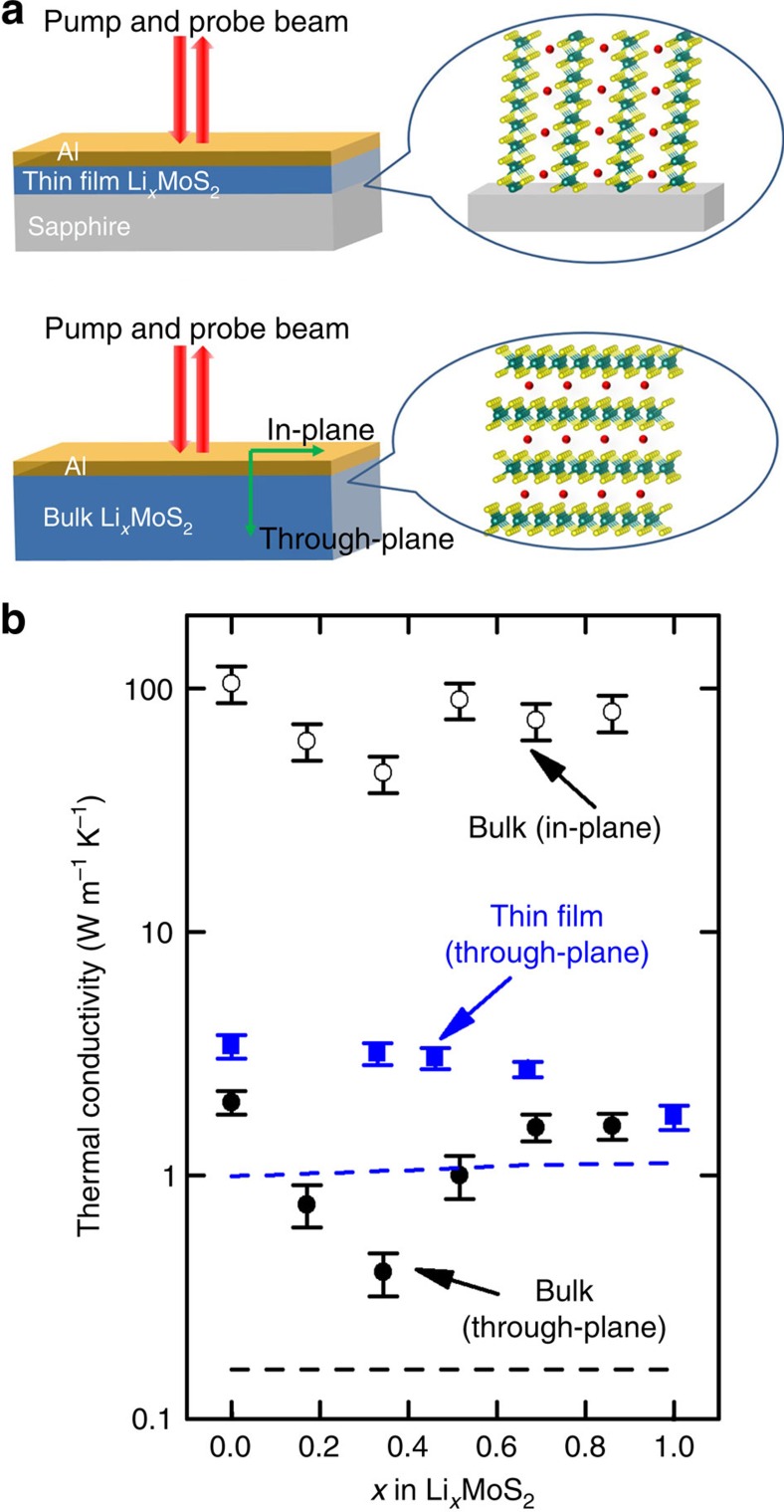
Thermal conductivity measurement of Li_*x*_MoS_2_ samples. (**a**) Schematics of bulk and thin-film Li_*x*_MoS_2_ samples for TDTR measurements. (**b**) Thermal conductivity of Li_*x*_MoS_2_ samples with different degrees of lithiation *x*. Blue squares: through-plane thermal conductivity of thin-film MoS_2_; black squares: through-plane thermal conductivity of bulk MoS_2_; black open squares: in-plane thermal conductivity of bulk MoS_2_. The minimum thermal conductivity for bulk and thin-film samples are plotted as black and blue dashed lines, respectively. The total uncertainties of the measured thermal conductivity are calculated by taking into account the systematic errors that propagate from uncertainties in the film thickness, laser spot size, and thermal properties of the transducer film and substrate.

MoS_2_ samples (schematics shown in Fig. 4a) were characterized by TDTR to determine the change in thermal conductivity caused by lithium ion intercalation. The TDTR data of both thin-film and bulk Li_*x*_MoS_2_ samples are presented as a function of *x* in Fig. 4b. The through-plane thermal conductivity of thin-film Li_*x*_MoS_2_ decreases monotonically from ≈3.4 W m^−1^ K^−1^ (*x*=0) to ≈1.7 W m^−1^ K^−1^ (*x*=1) with increasing lithium content. The through-plane thermal conductivity of bulk Li_*x*_MoS_2_ decreases first from ≈2.0 W m^−1^ K^−1^ (*x*=0) to ≈0.4 W m^−1^ K^−1^ (*x*=0.34) and then increases to ≈1.6 W m^−1^ K^−1^ (*x*=0.86). The in-plane thermal conductivity follows a similar trend, which decreases from ≈105 W m^−1^ K^−1^ (*x*=0) to ≈45 W m^−1^ K^−1^ (*x*=0.34) and then increases to ≈80 W m^−1^ K^−1^ (*x*=0.86).

To understand such a drastic decrease of thermal conductivity, we calculated the minimum thermal conductivity 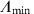 of Li_*x*_MoS_2_ using a simplified model by Cahill *et al*.^20^ at the high-temperature limit. The minimum thermal conductivity is 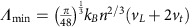, where *k*_*B*_ is the Boltzmann constant, *n* is the atomic density (atoms cm^−3^), *v*_*L*_ is the longitudinal speed of sound, and *v*_*t*_ is the transverse speed of sound^21^. Atomic densities of Mo and S atoms in Li_*x*_MoS_2_ thin films was measured by Rutherford backscattering spectrometry; *v*_*L*_ is measured by picosecond acoustics; *v*_*t*_ is measured by detecting surface acoustic waves using a phase-shift mask^22^. We accounted for the change of atomic density and longitudinal speed of sound due to lithiation. More details can be found in the ‘Methods' section and the Supplementary Methods.

In thin-film Li_*x*_MoS_2_ samples, the polycrystalline structure with randomly oriented columnar grains is transverse isotropic so that this conventional minimum thermal conductivity model can be applied. In the bulk Li_*x*_MoS_2_ samples, however, the strongly anisotropic structure introduces a significant phonon-focusing effect^23^, which suppresses the through-plane average group velocity due to the relatively high in-plane group velocity. We adopted the modified minimum thermal conductivity model recently proposed by Zhen *et al*.^41^ (equation S(7) in their Supplementary Material) and followed their procedure to calculate the minimum thermal conductivity of bulk Li_*x*_MoS_2_ (see details in Supplementary Note 1 with parameters shown in Supplementary Table 1).

If the measured lowest through-plane thermal conductivity of bulk or thin-film Li_*x*_MoS_2_ agrees with the predicted minimum thermal conductivity, the phonons are glass-like lattice vibrations in a disordered crystal at this composition. The calculated minimum thermal conductivity of bulk and thin-film Li_*x*_MoS_2_ is plotted in Fig. 4b as dashed lines. Both measured through-plane thermal conductivity of bulk and thin-film Li_*x*_MoS_2_ is higher than the predicted minimum thermal conductivity, which suggests that a significant fraction of the phonons in these samples are propagating modes.

### Interlayer distance characterization

**Figure 5:**
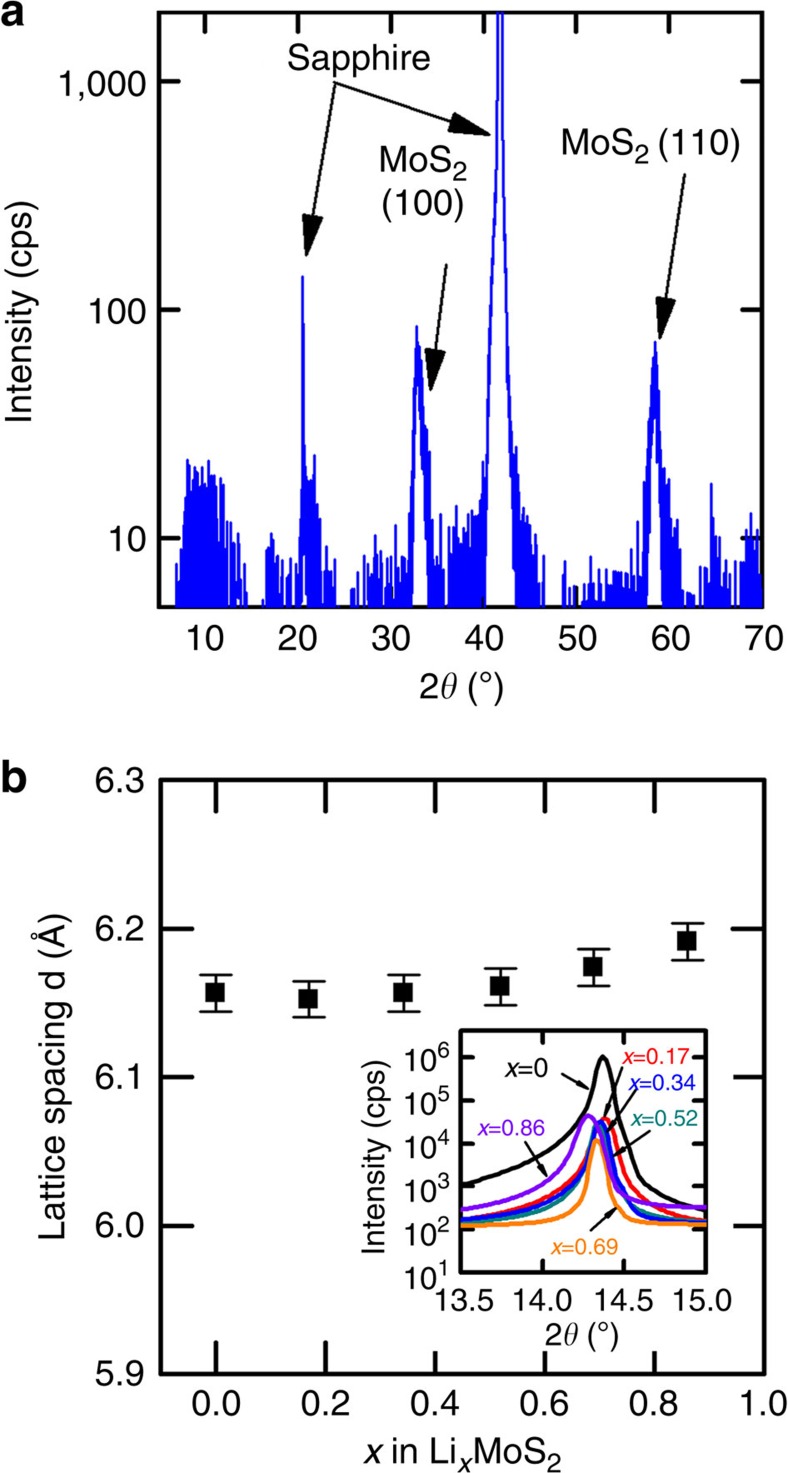
X-ray diffraction characterization of Li_*x*_MoS_2_ samples. (**a**) X-ray diffraction spectra for MoS_2_ thin-film samples on sapphire substrate. The two diffraction peaks coincide with the standard X-ray diffraction powder patterns of MoS_2_ (100) and (110). Therefore, the dominant lattice orientation in the MoS_2_ thin-film samples are (100) and (110). The diffraction peak at 32.8° and 58.5° corresponds to a lattice constant of 2.73 and 1.58 Å, respectively. (**b**) Lattice spacing *d* between MoS_2_ layers in bulk MoS_2_ samples. The error bars are calculated by taking into account the experimental errors and the systematic errors that propagate from uncertainties in the fitting of X-ray diffraction spectra. The inset plot is the change of MoS_2_ (002) peak position with *x* in Li_*x*_MoS_2_.

Large expansion in layer spacing along the *c*-axis is often observed after intercalation of large-diameter molecules or ions^11^,^24^. We collected X-ray diffraction spectra for bulk Li_*x*_MoS_2_ samples with different amount of lithium ion intercalation (0≤*x*≤0.86) to determine the change in layer spacing due to lithium ion intercalation. Samples were placed inside an air-tight sample holder with a beryllium (Be) window in an argon-filled glovebox before they were transferred out for X-ray diffraction characterization. Figure 5b shows that the (002) peak of the pristine MoS_2_ is located at 14.40^o^, and the peak position downshifts to 14.28^o^ for Li_0.86_MoS_2_. The corresponding layer spacing is 6.16 and 6.19 Å for pristine MoS_2_ and Li_0.86_MoS_2_, respectively, a 0.5% change in lattice constant. We estimate the uncertainty of the lattice spacing measurement as ≈0.6%. The weak dependence of lattice constant on lithium content is consistent with the fact the effective ionic radius of lithium ion^25^ (76 pm) is slightly smaller than the octahedral site in the van der Waals gap of in MoS_2_. If we assume an effective ionic radius of S^2−^ as 1.84 Å, and close packing of S^2−^ atoms, the radius of the octahedral site is 76 pm (ref. 25). Our measurement on the layer spacing of Li_0.86_MoS_2_ agrees with recent observations reporting on minimal change in interlayer distance of LiMoS_2_ (refs 26, 27).

### Elastic constants

**Figure 6:**
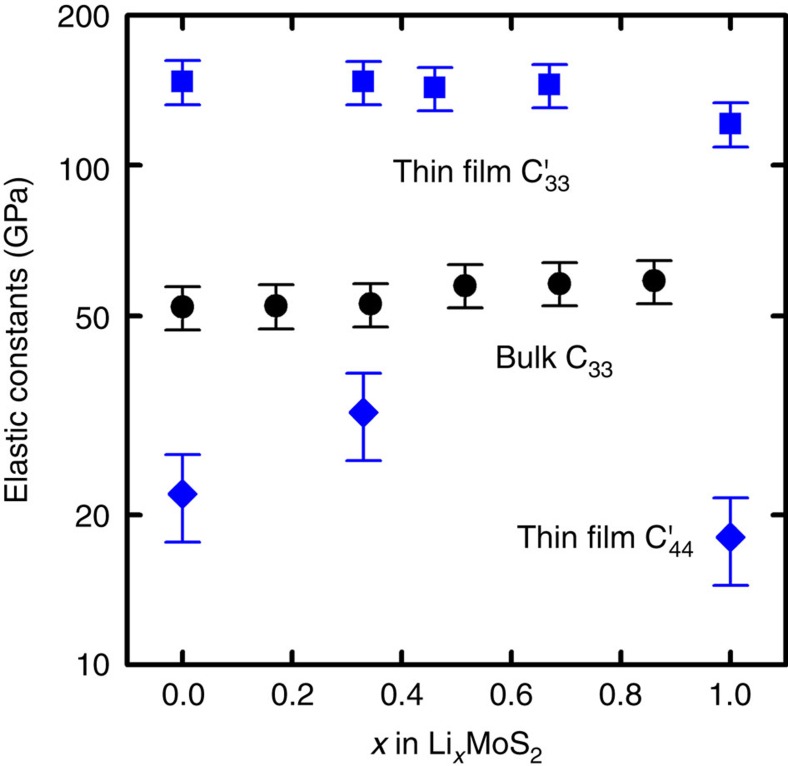
Effective elastic constant of thin-film Li_*x*_MoS_2_ with different degrees of lithiation. 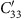
 (square) decreases from 147 GPa (*x*=0) to 121 GPa (*x*=1). 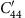 (diamond) increases from 22 GPa (*x*=0) to 32 GPa (*x*=0.34) and then decreases to 18 GPa (*x*=1). As comparison, the *C*_33_ of bulk Li_*x*_MoS_2_ samples (circle) are also plotted. *C*_33_ gradually changes from 52 GPa (*x*=0) to 58 GPa (*x*=0.86) with a transition point at *x*≈0.34. The error bars are calculated by taking into account the experimental errors and the systematic errors that propagate from uncertainties in the Al film thickness, Li_*x*_MoS_2_ film density and the input elastic constants.

We further measured elastic properties using pump-probe techniques to help understand the thermal conductivity change in our samples. Polycrystalline MoS_2_ thin films with vertically aligned basal planes are transverse isotropic, which has five effective independent averaged elastic constants: 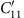, 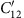, 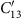, 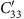 and 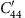. Figure 6 plots the effective 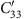 and 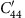 elastic constants of thin-film Li_*x*_MoS_2_. The elastic constant 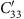 of Li_*x*_MoS_2_ thin film decreases from 147 GPa (*x*=0) to 121 GPa (*x*=1). Even though the density of Li_*x*_MoS_2_ thin films increases by 11% from *x*=0 to *x*=1, the decrease of longitudinal speed of sound from 5,720 ms^−1^ to 4,930 ms^−1^ (measured by picosecond acoustics) dominates the decrease of 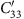. The elastic constant 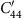 increases from 22 GPa (*x*=0) to 32 GPa (*x*=0.34) and then decreases to 18 GPa (*x*=1). We do not yet understand the trend of the elastic constants of thin-film Li_*x*_MoS_2_ changing with *x*. One possible reason is the combined effect of increasing binding energy due to the intercalation of lithium ions and increasing structural and compositional disorder (for example, point defects and mixture of phases). More measurement details are described in the ‘Methods' section and Supplementary Methods.

The elastic constants of bulk Li_*x*_MoS_2_ samples *C*_33_ are also plotted in Fig. 6. *C*_33_ gradually changes from 52 GPa (*x*=0) to 58 GPa (*x*=0.86) with a transition point at *x* ≈0.34, which suggests a phase transition in bulk Li_*x*_MoS_2._

### Raman spectroscopy

We used Raman spectroscopy to further characterize lithiation-induced structural and compositional disorder of bulk Li_*x*_MoS_2_ to gain more insights about other phonon scattering mechanisms that could lead to the thermal conductivity change. All bulk Li_*x*_MoS_2_ samples except pristine MoS_2_ were loaded inside the glovebox into a home-made air-free sample holder sealed by O-ring and screw-on connectors and measured through the glass window of the holder. As shown in Fig. 2c,d, the intensity of the low-frequency peak at ∼32 cm^−1^ corresponding to the 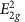 shear mode in 2H-MoS_2_ decreases as the degree of lithiation (*x*) increases. The decreasing peak intensity of 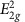
mode is attributed to the increasing stacking disorder resulting from the lithium ion intercalation. In the 2H to 1T phase transition, the stacking of Mo atom planes changes from ABA (two molecular layers per unit cell) to AA (one molecular plane per unit cell)^28^,^29^. As the atomic structure of the MoS_2_ layer changes from prismatic *aBa* (2H) structure to octahedral *aBc* (1T) (upper letters correspond to Mo planes, lowercase letters correspond to the S planes)^30^, the high-frequency 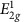 and *A*_1*g*_ modes redshift from 383 to 377 cm^−1^ and 408 to 402 cm^−1^, respectively, as the lithium content increases from *x*=0 to *x*=0.86. The observed redshift is mainly attributed to the intra-layer shift of the S atom during the 2H to 1T phase change^31^.

## Discussion

Lithium ion intercalation into van der Waals gap could change the thermal transport of MoS_2_ due to several distinct mechanisms. First, the 2D layer spacing might change as a result of lithiation^10^,^11^,^19^. In addition, a recent theoretical work found that intercalated lithium ions enhances the binding energy through orbital hybridizations between cations (lithium ions) and anions (MoS_2_) (ref. 32). Second, the intercalated lithium donates electrons to MoS_2_, which changes the oxidation states of Mo, and hence the electronic properties of MoS_2_ (refs 33, 34). Finally, intercalation drives a phase transition from the semiconductor 2H phase to the metallic 1T phase^19^.

The increase of electrical conductivity caused by the phase transition from the semiconductor 2H to metallic 1T phase cannot explain the changes in the in-plane thermal conductivity of bulk Li_*x*_MoS_2_. A recent study shows the in-plane electrical conductivity of the metallic phase (1T) LiMoS_2_ is ≈300 S cm^−1^, ∼500 times that of the semiconducting phase (2H) MoS_2_ (ref. 35). The corresponding electronic contribution to the total thermal conductivity, predicted by the Wiedemann–Franz law, is only 0.2 W m^−1^ K^−1^ in LiMoS_2_. Such a small increase cannot be the predominant mechanism to account for the in-plane thermal conductivity change in bulk Li_*x*_MoS_2_ from 45 to 80 W m^−1^ K^−1^ for *x*=0.34 to 0.86.

We applied the Leibfried–Schlomann (LS) equation to evaluate the effect of changes in lattice spacing, elastic constants and mass density on the thermal conductivity of Li_*x*_MoS_2_. The LS equation only considers changes in three-phonon scattering rates and takes the form 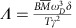, where 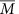 is the mass of a unit cell, *δ*^3^ corresponds to atomic volume, *T* is temperature, *γ* is the Grüneisen parameter for the relevant direction of heat transfer, *ω*_*D*_ is the Debye frequency for the relevant direction of heat transfer, and *B* is an amalgam of physical constants^36^. Using the Debye frequency defined as *ω*_*D*_=*v*_*D*_*k*_*D*_, where *k*_*D*_ is the Debye cutoff vector, *v*_*D*_ is the speed of sound, we can express the thermal conductivity 
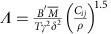
, where *B′* is a constant, *C*_*ij*_ is the elastic constant and *ρ* is the density. Considering a 4% increase in atomic mass of the unit cell, 11% increase in density, 18% decrease in the effective elastic constant 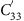 and 0.5% increase in lattice spacing for Li_*x*_MoS_2_ thin films from *x*=0 to 1, the LS equation predicts a 33% decrease in the through-plane thermal conductivity, if we assume that the change in Grüneisen parameter is negligible. Since TDTR measurement shows that through-plane thermal conductivity of thin-film Li_*x*_MoS_2_ decreases continuously from ≈3.4 to ≈1.7 W m^−1^ K^−1^ from *x*=0 to 1, we conclude that the softening of the lattice is a significant contribution to the reduction in thermal conductivity in Li_*x*_MoS_2_ thin films.

Similarly for the bulk samples, if we consider the changes of elastic constants, lattice spacing, density and atomic mass in a unit cell to be 11%, 0.5%, 3.3%, 3.8%, respectively from *x*=0 to *x*=0.86, the through-plane thermal conductivity is estimated to increase by ≈14%. Under the same assumptions, the through-plane thermal conductivity of Li_*x*_MoS_2_ should increase by ≈5% from *x*=0 to *x*=0.34. Such a small change and different trends indicate the lattice expansion and elastic constant change cannot explain the pronounced change in the through-plane thermal conductivity that we observed in bulk Li_*x*_MoS_2_ samples.

Interestingly, Raman spectroscopy of bulk Li_*x*_MoS_2_ samples revealed that the *A*_1*g*_ mode peak splits into two peaks at 402 and 408 cm^−1^, while the *E*_2*g*_^2^ peak slightly broadens in the *x*=0.34 sample suggesting the co-existence of 2H and 1T phases. The peak splitting was related to the Davydov pairs of the optical phonon branches due to the splitting of intra-layer modes caused by the interlayer interaction when the lithium content is below *x*=0.34 (ref. 29). We do not yet have a good understanding why the split of 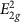 peak is not clearly observed in the *x*=0.34 sample but it may be related to the intermediate atomic structure in the not-completed phase-change process. Both high- and low-frequency Raman spectra provide evidence of increasing stacking disorder and mixture of phases during lithiation.

As a result of co-existence of two phases and increasing stacking disorder, phonon-boundary scattering increases and thermal conductivity in both through-plane and in-plane directions decrease until the largest degree of disorder is reached at *x*≈0.34. As intercalation proceeds, the bulk MoS_2_ sample becomes increasingly dominated by the 1T phase (0.34<*x*<0.86) and the phonon-boundary scattering rate decreases, and hence the thermal conductivity starts to increase again.

Our most striking observation is that the thermal anisotropy ratio of the bulk Li_*x*_MoS_2_ increases from 52 (*x*=0) to 110 (*x*=0.34) with increasing disorder. This trend of thermal anisotropy ratio change with respect to the structural defects or disorder is indeed counter-intuitive and beyond any previous works. For example, a recent study of graphene oxide films by Renteria *et al*.^14^ shows an increase of thermal anisotropy ratio attributed to decreasing disorder after annealing. Luckyanova *et al*.^13^ found that the thermal anisotropy ratio decreases with interface atomic mixing in superlattices. The thermal anisotropy ratio of a disordered WSe_2_ crystal, ≈30, is similar to the anisotropy ratio of well-ordered WSe_2_ crystals^37^,^38^. Our measurements of lithiated MoS_2_ bulk crystal demonstrate that lithiation-induced stacking disorder and phase transition can increase the thermal anisotropy ratio.

Phonon–phonon and phonon-boundary scattering are the two possible major scattering mechanisms in bulk Li_*x*_MoS_2_. As discussed above, we estimated the change of the intrinsic lattice thermal conductivity that is limited by phonon–phonon interactions due to the changes in elastic constants and atomic densities. The predicted change in the intrinsic phonon–phonon scattering rates is small and we therefore exclude changes in phonon–phonon scattering as the dominant mechanism in lithiated bulk Li_*x*_MoS_2_. The combination of our Raman spectroscopy data—and the pronounced change in the thermal conductivity of bulk samples—suggest that phonon scattering in bulk Li_*x*_MoS_2_ is dominated by phonon-boundary scattering due to stacking disorder created by the phase transition.

In highly anisotropic layered materials, boundary-limited phonon mean-free paths in the in-plane and through-plane directions can differ by orders-of-magnitude due to the combination of phonon-focusing effects and divergent length scales of disorder in the in-plane versus through-plane directions. When boundary scattering dominates the phonon mean-free paths, phonon-focusing produces anisotropic phonon mean-free path even in cubic crystals^39^. A recent modelling study^40^ demonstrates that thin films of graphite can maintain their high in-plane thermal conductivity even for film thickness as small as 10 nm. In other words, in highly anisotropic crystals, boundary scattering along the *c*-axis has only a minimal effect on the *ab*-plane thermal conductivity. Further experimental evidence of this effect comes from a recent experimental study^41^ of CVD-grown graphite thin films where the phonon mean-free paths are different by more than an order of magnitude: the in-plane phonon mean-free path is ≈300 nm and the through-plane mean-free path is <20 nm.

Although a sophisticated phonon transport model is needed to rigorously analyse how different phonon branches are affected by boundaries and other forms of disorder, we can estimate how the boundary-limited phonon mean-free paths in the *c*-axis *L*_*c*_ and in the ab-plane *L*_*ab*_ change with *x* by comparing our measured thermal conductivities with previous studies. We compare the measured in-plane thermal conductivities of bulk Li_*x*_MoS_2_ with the thermal conductivity of single-layer MoS_2_ predicted by a first-principle based Boltzmann transport equation calculation as a function of phonon-boundary scattering^42^. This comparison suggests *L*_*ab*_ decreases from >1 μm to ≈200 nm for *x*=0–0.34.

Similarly, the comparison between our measured through-plane thermal conductivity of bulk Li_*x*_MoS_2_ and that predicted by first-principle based Boltzmann transport equation calculations suggests *L*_*c*_≈200 nm at *x*=0 (ref. 43). The lowest through-plane thermal conductivity in our bulk samples is comparable to that of turbostratic nano-crystalline MoS_2_ thin films deposited by magnetron sputtering^36^. In the turbostratic thin-film samples, the length of coherent stacking is unknown but should be less than the grain size ≈5 nm (ref. 44). The similar stacking disorder in our bulk samples Li_*x*_MoS_2_ suggests the *L*_*c*_<5 nm at *x*=0.34. A plausible explanation for this pronounced phonon mean-free path difference is that the lithium ions diffuse more easily along the in-plane direction compared with the through-plane direction. As a result, the density of disorder caused by lithium ion intercalation is less pronounced along the in-plane direction, Both *L*_*c*_ and *L*_*ab*_ reach their minimum values with the maximum mixing of the two phases at *x*=0.34.

Finally, we noticed that the through-plane thermal conductivity of thin-film Li_*x*_MoS_2_ sample is much lower than the in-plane thermal conductivity of the bulk Li_*x*_MoS_2_ sample, and the trends in through-plane thermal conductivity as a function of *x* are different for bulk and thin-film samples. Compared with bulk MoS_2_, the basal planes in Li_*x*_MoS_2_ thin films contain high density of defects, especially point defects and oxygen impurities introduced during the chemical-vapour growth process of the thin sample. Defects can significantly reduce the thermal conductivity of 2D materials by enhancing phonon scattering^45^,^46^,^47^. Although the semiconductor 2H to metallic 1T phase transition also occurs in the thin-film sample as indicated by the plateau in the discharge curve, unlike the bulk sample, the thermal conductivity of defective thin-film Li_*x*_MoS_2_ is more sensitive to lattice softening than stacking disorder^47^. On the basis of our elastic constants measurement results and estimation by LS equation, we attribute the decreasing trend of thermal conductivity in Li_*x*_MoS_2_ thin films with increasing lithium content *x* to the softening of the lattice and the increased phonon-boundary scattering at the grain or phase boundaries^48^.

We investigated the anisotropic thermal transport in bulk crystal and CVD-grown thin-film MoS_2_ samples with different amounts of lithium ion intercalation. We demonstrated that intercalation impacts thermal transport in bulk and thin-film samples differently, depending on the crystalline quality of the 2D structure. In addition, we found that lithiation tends to reduce the phonon mean-free path more along the through-plane direction in Li_*x*_MoS_2_, rather than the in-plane direction, until the largest degree of disorder is reached at *x*≈0.34, leading to a significant increase in the thermal anisotropy ratio. This work provides insight on the impact of structural and compositional changes (for example, disorder, layer spacing and interfaces) on 2D materials applications, where thermal management is crucial.

## Methods

### Synthesis of thin-film MoS_2_

MoS_2_ thin films were grown on sapphire in a horizontal quartz tube furnace (Lindberg/Blue M 1,100 °C Tube Furnace with 1 in diameter tube). The substrate was pre-deposited with 70 nm Mo thin film, which shows electrical conductivity of about 4.8 × 10^5^ S cm^−1^. The Mo film was then placed in the centre of the tube furnace and S powder (Sigma Aldrich) was loaded in a ceramic crucible on the upstream cooler zone of the tube furnace. The temperature of the S was kept between 200 and 250 °C when the centre of the tube furnace reaches 750 °C. The tube was initially purged with Ar gas (100 sccm) for 10 min to remove oxygen. During the chemical-vapour growth of MoS_2_ thin film, Ar gas flow rate was kept at 100 sccm, and the temperature of the tube furnace was quickly increased to 750 °C in 15 min and then held for 20 min, followed by natural cooling down.

### Electrochemical intercalation of lithium

The thin-film and bulk MoS_2_ samples were assembled into battery configuration inside an argon-filled glovebox for lithium ion intercalation. Lithiation of thin-film MoS_2_ was performed in a glass vial. The thin-film MoS_2_ and a piece of lithium foil were used as the cathode and anode, respectively, and 1.0 M LiPF6 in 30:70 (vol%) ethylene carbonate/dimethyl carbonate (Sol-Rite) as the electrolyte. The thin-film sample was wrapped around by a piece of stainless steel foil, which was used as the electrical contact. The discharge current was 14 μA. The lithium ion intercalation of the bulk MoS_2_ was carried out using 2025 coin cells, with MoS_2_ as the cathode, lithium foil as the anode, 1.0 M LiPF6 in 30:70 (vol%) ethylene carbonate/dimethyl carbonate as electrolyte, and a Celgard 2400 separator. The discharge current used for the bulk MoS_2_ was 10 μA. After the discharge process, all samples were relaxed for days before they were cleaned by diethyl carbonate (anhydrous, Sigma Aldrich) inside the glovebox to remove the electrolyte left on the sample surfaces. Samples were sealed in air-tight aluminium pouches before they were transferred out of the glovebox and mailed from Ann Arbor, MI to Urbana, IL for sputtering deposition of Al or NbV for TDTR measurements.

### TDTR measurements

TDTR was used to measure the thermal conductivity of bulk and thin-film intercalated MoS_2_. The experimental setup and model details on TDTR can be found elsewhere^49^,^50^. All the other details are shown in Supplementary Methods.

Before the TDTR measurements, metal thin films (Al or NbV) were deposited on the samples by magnetron sputtering. Samples were exposed to air for only 3–5 s before the process chamber was pumped down. We measured through-plane thermal conductivity of MoS_2_ at *f*=9.8 MHz, with a *1/e*^*2*^ radius of the focused laser beams *w*_0_=11.7 μm. The thermal conductivity of MoS_2_ thin films reported in this work is the apparent (or effective) thermal conductivity of the thin film, including the two interfacial thermal resistances between MoS_2_ and the neighbouring materials, besides the intrinsic thermal resistance of the film. The sensitivity of the TDTR data to the in-plane thermal conductivity of Li_*x*_MoS_2_ thin film is small, which makes this in-plane thermal conductivity challenging to measure.

We measured through-plane thermal conductivity of bulk MoS_2_ at *f*=9.8 MHz with *w*_0_=11.7 μm. The thermal conductivity of bulk MoS_2_ and the interfacial thermal conductance between Al and MoS_2_ were fitted. The in-plane thermal conductivity of bulk MoS_2_ was measured using the beam-offset TDTR method as detailed in ref. 6, at *f*=1.1 MHz with *w*_0_≈27 μm. NbV transducer was used in this measurement, whose thermal properties were characterized in ref. 51. The total uncertainties of the measured thermal conductivity are calculated by taking into account the systematic errors that propagate from uncertainties in the film thickness, laser spot size and thermal properties of the transducer film and substrate. We have tried to use a 65 nm-thick NbV thin film as the metal transducer to measure in-plane thermal conductivity *Λ* of MoS_2_ thin film by TDTR method. However, due to the relatively low thermal conductance of the film, that is, 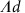, where *d* is the thickness of the thin film the in-plane heat flow in metal transducer and the Sapphire substrate instead of the MoS_2_ thin film dominates the lateral heat flow which lead to a low sensitivity to the thin film in-plane thermal conductivity in TDTR measurement. The thermal conductivity measurements are performed at different locations on our samples to confirm the homogeneous distribution of lithium.

### Elastic constants measurement

The elastic constants of the Li_*x*_MoS_2_ thin films were measured using pump-probe techniques. The polycrystalline MoS_2_ thin film with vertically aligned basal planes is transverse isotropic, which has five effective independent averaged elastic constants: 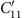, 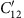, 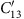, 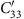 and 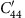. The error bars, ∼20%, are calculated by taking into account the experimental errors and the systematic errors that propagate from uncertainties in the Al film thickness, Li_*x*_MoS_2_ film density and the input elastic constants.

Similarly, the elastic constants of bulk Li_*x*_MoS_2_ (*C*_33_) were calculated from 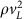, where *ρ* is calculated based on literature values of bulk MoS_2_ samples (5.06 g cm^−3^) and the degree of lithiation *x*, *ρ*=5.06(160+7*x*)/160. We deposited ≈10 nm NbV on the bulk Li_*x*_MoS_2_ samples and used picosecond interferometry^52^ to determine the longitudinal speed of sound *v*_*L*_. In the picosecond interferometry, the Brillouin scattering frequency *f*_B_ is related to the longitudinal speed of sound *v* by *f*_B_=2*nv*_*L*_/*λ*, where *n* is the index of refraction of the sample and *λ* is the laser wavelength. We used the literature value of *n*≈4.7 at *λ*=785 nm in this calculation^53^. This measurement of picosecond interferometry uses the same experiment setup as TDTR. All the other details are shown in Supplementary Methods.

### Raman spectroscopy measurement

The Raman spectroscopy measurements were performed using an Acton Insight spectrometer (Princeton Instruments). The excitation wavelength is 488 nm from a Spectra-Physics Cyan (CDRH) solid-state laser. A power of ≈1 mW is used to avoid excessive sample heating. We used an optical configuration similar to that d multilayer graphene^54^; the laser plasma lines are removed using a BragGrate bandpass filter (OptiGrate), while the Rayleigh line is suppressed using three BragGrate notch filters (OptiGrate) in series each with an optical density 3 and a spectral bandwidth 5–10 cm^−1^. The backscattered signal was collected through a 20 × objective (numerical aperture=0.4) with laser spot size ≈10 μm at the sample surface and dispersed by a 1,200 g mm^−1^ grating with a spectral resolution ≈2 cm^−1^.

### Additional structural analysis

TEM specimens of the thin-film MoS_2_ samples were prepared using a FEI FIB200 Focused Ion Beam system. The bulk TEM specimens were prepared by hand grinding and ultrasonication in ethanol. TEM was performed using a Hitachi HD 2300 STEM. X-ray diffraction was performed using a Rigaku SmartLab x-ray diffractometer.

### Data availability

The data supporting the main findings of this study are available from the corresponding authors on request.

## 

## Additional information

**How to cite this article:** Zhu, G. *et al*. Tuning thermal conductivity in molybdenum disulfide by electrochemical intercalation. *Nat. Commun.* **7,** 13211 doi: 10.1038/ncomms13211 (2016).

## Supplementary Material

Supplementary Information Supplementary Table 1, Supplementary Note 1, Supplementary Methods and Supplementary References
