# Supplementary material for: Tuning thermal conductivity in molybdenum disulfide by electrochemical intercalation
Source: Nat Commun. 2016 Oct 21;7:13211. doi: 10.1038/ncomms13211 (PMC5078741; doi:10.1038/ncomms13211)
Supplement: Supplementary Information — Supplementary Table 1, Supplementary Note 1, Supplementary Methods and Supplementary References [file ncomms13211-s1.pdf]

Supplementary Table 1. Parameters for estimating minimum thermal conductivity in MoS_2 crystal. The three polarizations (TL1, TL2, and TA) are named following the isoenergy-decomposition process described in Ref. 1.

	**Unit**	***ab*-plane**	***c*-axis**
$\mathbf{v_{TL1}}$	$m\ s^{-1}$	6850	1938
$\mathbf{v_{TL2}}$	$m\ s^{-1}$	1938	3206
$\mathbf{v_{TA}}$	$m\ s^{-1}$	5372	1938
$\mathbf{k_{max}}$	$10^{10}\ m^{-1}$	2.34	0.51
$\boldsymbol{\theta_{D,\ TL1}}$	K	1220	75
$\boldsymbol{\theta_{D,\ TL2}}$	K	345	124
$\boldsymbol{\theta_{D,\ TA}}$	K	956	75

Supplementary Note 1

Minimum thermal conductivity calculation

We follow the method described in Ref. 1 to calculate the minimum thermal conductivity in the *c*-axis and *ab*-plane for MoS_2 crystal. To calculate minimum thermal conductivity in the *c*-axis, we used the following equation S(1), equation (S7) from the Supporting Information of Ref. 1,

$$\kappa_{\min-c,Layered} = \sum_{pol} \frac{v_c}{6\pi v_{ab}^2} \frac{k_B^3}{\hbar^2} \left[\int_0^{x_{D,c}} \frac{T^2 x^3 e^x}{\left(e^x - 1\right)^2} dx + \frac{\theta_{D,c}^3}{T} \int_{x_{D,c}}^{x_{D,ab}} \frac{e^x}{\left(e^x - 1\right)^2} \left(\frac{\theta_{D,ab}^2 - \left(Tx\right)^2}{\theta_{D,ab}^2 - \theta_{D,c}^2} \right)^{\frac{3}{2}} dx \right], \qquad S(1)$$

where $x = \hbar\omega / k_B T$. We took the lattice constants (a, b, c), density, and all the elastic constants of MoS_2 crystal from Ref.2. The Debye temperature of the *c*-axis $\theta_{D,c}$ was calculated using

$$\theta_{D,c} = \frac{\hbar v_c k_{\max,c}}{k_B}, \qquad S(2)$$

where $\hbar$ is the Planck constant, k_B is the Boltzmann constant, $k_{\max,c}$ is the maximum wave vector in the chain axis direction. The Debye temperatures in the other two directions were calculated similarly. The group velocities in each polarization were estimated from the elastic constants (C_{11}=238 GPa, C_{33}= 52 GPa, C_{44}= 19 GPa, C_{66}= 146 GPa, C_{13}=13 GPa) and the density ρ=5.06 g/cm^3 of MoS_2 crystal using $\sqrt{C_{ij}/\rho}$. Supplementary Table 1 shows all the parameters we used in this calculation.

Similarly, we used the following equation S(1), equation (S9) from the Supporting Information of Ref. 1 to calculate the minimum thermal conductivity in the *ab*-plane,

$$\kappa_{\min-ab,Layered}=\sum_{pol}\frac{1}{6\pi v_c}\frac{k_B^3}{\hbar^2}\left[\begin{array}{l}\int_0^{x_{D,c}}\frac{T^2x^3e^x}{\left(e^x-1\right)^2}dx+\frac{3}{2}\theta_{D,c}\int_{x_{D,c}}^{x_{D,ab}}\frac{Tx^2e^x}{\left(e^x-1\right)^2}\left(\frac{\theta_{D,ab}^2-\left(Tx\right)^2}{\theta_{D,ab}^2-\theta_{D,c}^2}\right)^{\frac{1}{2}}dx\\-\frac{1}{2}\frac{\theta_{D,c}^3}{T}\int_{x_{D,c}}^{x_{D,ab}}\frac{e^x}{\left(e^x-1\right)^2}\left(\frac{\theta_{D,ab}^2-\left(Tx\right)^2}{\theta_{D,ab}^2-\theta_{D,c}^2}\right)dx\end{array}\right]. \quad S(3)$$

The minimum thermal conductivity in the *c*-axis for a MoS_2 crystal is 0.16 W m^{-1} K^{-1}, with contributions from TL1, TL2, and TA modes 0.004 W m^{-1} K^{-1}, 0.151 W m^{-1} K^{-1}, and 0.006 W m^{-1} K^{-1}, respectively. The minimum thermal conductivity in the *ab*-plane for a MoS_2 crystal is 1.0 W m^{-1} K^{-1}, with contributions from TL1, TL2, and TA modes 0.5 W m^{-1} K^{-1}, 0.1 W m^{-1} K^{-1}, and 0.4 W m^{-1} K^{-1}, respectively.

Supplementary Methods

Thermal Conductivity Measurements

TDTR was used to measure the thermal conductivity of bulk and thin film intercalated MoS_2. In TDTR, a mode-locked Ti:sapphire laser produces a train of pulses at a repetition rate of 80 MHz. The laser beam is split into pump and probe beams. A mechanical delay stage is used to change the optical path difference between the pump and probe beams before they are focused onto the sample surface through an objective lens. The pump beam is modulated at frequency *f* so that the thermoreflectance change at the sample surface can be detected by the probe beam through lock-in detection. The ratio of the in-phase and out-of-phase signal from the lock-in is then fit to a thermal diffusion model. Further details on TDTR can be found elsewhere.[3, 4]

Prior to the TDTR measurements, metal thin films (Al or NbV) were deposited on the samples by magnetron sputtering. Samples were exposed to air for only three to five seconds before the process chamber was pumped down. We measured through-plane thermal conductivity of MoS_2 at f= 9.8 MHz, with a $1/e^2$ radius of the focused laser beams $w_0 = 11.7\ \mu m$. In the fitting, the heat capacities of Al, NbV, and sapphire are adopted from literature values.[5, 6] The thickness of Al thin film was obtained from picosecond acoustics using a longitudinal speed of sound 6.42 nm ps^{-1}.[7] The thickness of the MoS_2 thin films was measured by atomic force microscopy (AFM). The thermal conductivity of the Al thin film was calculated using the Wiedemann-Franz law and the electrical resistance of the same transducer layer deposited on a ≈315 nm SiO_2 on Si reference sample. The thermal conductivity of sapphire substrate, 32 $Wm^{-1}K^{-1}$, was measured using the same Al transducer. The thermal conductivity of MoS_2 thin films reported in this work is the apparent (or effective) thermal conductivity of the thin film,

including the two interfacial thermal resistances between MoS_2 and the neighboring materials, besides the intrinsic thermal resistance of the film. The sensitivity of the TDTR data to the in-plane thermal conductivity of Li_xMoS_2 thin film is small, which makes this in-plane thermal conductivity challenging to measure.

We measured through-plane thermal conductivity of bulk MoS_2 at f= 9.8 MHz with $w_0 = 11.7\ \mu m$. The thermal conductivity of bulk MoS_2 and the interfacial thermal conductance between Al and MoS_2 were fitted. The in-plane thermal conductivity of bulk MoS_2 was measured using the beam-offset TDTR method as detailed in ref. 8 , at f= 1.1 MHz with $w_0 \approx 2.7\ \mu m$. NbV transducer was used in this measurement, whose thermal properties were characterized in ref. [9]. The total uncertainties of the measured thermal conductivity are calculated by taking into account the systematic errors that propagate from uncertainties in the film thickness, laser spot size, and thermal properties of the transducer film and substrate. We have tried to use a 65 nm-thick NbV thin film as the metal transducer to measure in-plane thermal conductivity Λ of MoS_2 thin film by TDTR method. However, due to the relatively low thermal conductance of the film, i.e., Λd , where d is the thickness of the thin film the in-plane heat flow in metal transducer and the Sapphire substrate instead of the MoS_2 thin film dominates the lateral heat flow which lead to a low sensitivity to the thin film in plane thermal conductivity in TDTR measurement. The thermal conductivity measurements are performed at different locations on our samples to confirm the homogeneous distribution of lithium.

Elastic constants measurements

The elastic constants of the Li_xMoS_2 thin films were measured using pump-probe techniques. The polycrystalline MoS_2 thin film with vertically-aligned basal planes is transverse

isotropic, which has five effective independent averaged elastic constants: C'_{11}, C'_{12} , C'_{13}, C'_{33}, and C'_{44}. C'_{33} is calculated from ρv_L^2, where the longitudinal speed of sound $v_L = 2d/t$, where t was measured using the longitudinal acoustic echoes in a TDTR measurement. Film thickness d was measured by atomic force microscope (AFM). The film density was calculated by $\rho = nM/(dN_A)$, where M is the molecular weight of Li_xMoS_2 and N_A is the Avogadro constant. We used Rutherford backscattering spectrometry (RBS) measurement to determine the areal atomic density n_1 (atoms m^{-2}) of Mo and S atoms in Li_xMoS_2 thin films and calculated the total areal atomic density n (atoms m^{-2}) of Li_xMoS_2.

The C'_{44} of Li_xMoS_2 thin films is determined from the velocity of surface acoustic wave (SAW) v_{SAW}, which is generated and detected in the pump-probe experiment using an elastomeric phase-shift mask made of PDMS.[10] The thickness of the Al transducer used in the SAW measurements is ≈85 nm. We calculated v_{SAW} of the tri-layer structure (Al/MoS_2/Sapphire) numerically using a Green's function method.[11] The inputs to the calculation are elastic constants, density and thickness of each layer. The Al layer ($C_{11} = 108\,\mathrm{GPa}$, $C_{12} = 63\,\mathrm{GPa}$, $C_{44} = 28.3\,\mathrm{GPa}$, $\rho = 2.7\,\mathrm{g\,cm^{-3}}$)[10] is a (111) textured film and the sapphire substrate ($C_{11} = 497\,\mathrm{GPa}$, $C_{12} = 163\,\mathrm{GPa}$, $C_{33} = 501\,\mathrm{GPa}$, $C_{44} = 147\,\mathrm{GPa}$, $\rho = 3.95\,\mathrm{g\,cm^{-3}}$)[12] has its basal plane facing up. Under the assumption that the c-axis of MoS_2 was randomly oriented in plane and the grains were much smaller than spot size (~10 µm), we calculated the effective elastic constants of the transverse isotropic MoS_2 film by averaging from the elastic constants of bulk crystal[13] using derivation from Ref. 14. For our typical sample structure Al(100nm)/MoS_2(200nm)/Sapphire, the sensitivity of v_{SAW} to C'_{33} and C'_{44} are 0.19 and 0.12,

respectively, while the sensitivity to C'_{11}, C'_{12}, C'_{13} are 0.02, -0.001 and 0.01, respectively. The MoS2 film is modeled using the calculated C'_{11}, C'_{12}, C'_{13}, and measured C'_{33}. C'_{44} was adjusted in the calculation to match the calculated v_{SAW} to the measurement data. The error bars, approximately 20%, is calculated by taking into account the experimental errors and the systematic errors that propagate from uncertainties in the Al film thickness, Li_xMoS_2 film density, and the input elastic constants.

Similarly, the elastic constants of bulk Li_xMoS_2 (C_{33}) were calculated from ρv_L^2, where ρ is calculated based on literature values of bulk MoS_2 samples (5.06 g/cm^3) and the degree of lithiation x, $\rho = 5.06(160 + 7x)/160$. We deposited ≈10 nm NbV on the bulk Li_xMoS_2 samples and used picosecond interferometry[15] to determine the longitudinal speed of sound v_L. In the picosecond interferometry, the Brillouin scattering frequency f_B is related to the longitudinal speed of sound *v* by $f_B = 2nv_L/\lambda$, where *n* is the index of refraction of the sample and λ is the laser wavelength. We used the literature value of *n*≈4.7 at λ=785 nm in this calculation.[16] This measurement of picosecond interferometry uses the same experiment setup as TDTR.

Supplementary References

1. Chen, Z. & Dames, C. An anisotropic model for the minimum thermal conductivity. *Appl. Phys. Lett.* **107**, 193104 (2015).
2. Wakabayashi, N., Smith, H.G. & Nicklow, R.M. Lattice dynamics of hexagonal MoS2 studied by neutron scattering. *Phys. Rev. B* **12**, 659-663 (1975).
3. Cahill, D.G. Analysis of heat flow in layered structures for time-domain thermoreflectance. *Rev. Sci. Instrum.* **75**, 5119-5122 (2004).
4. Liu, J. et al. Simultaneous measurement of thermal conductivity and heat capacity of bulk and thin film materials using frequency-dependent transient thermoreflectance method. *Rev. Sci. Instrum.* **84**, 034902 (2013).
5. Ditmars, D.A., Plint, C.A. & Shukla, R.C. Aluminum. I. Measurement of the relative enthalpy from 273 to 929 K and derivation of thermodynamic functions for Al(s) from 0 K to Its melting point. *International Journal of Thermophysics* **6**, 499-515 (1985).
6. O. Madelung, U. Rössler & Schulz, M. (eds.) Silicon (Si), Debye temperature, heat capacity, density, hardness, melting point, Vol. 41A1b. (Springer-Verlag, 2002).
7. Cahill, D.G. & Watanabe, F. Thermal conductivity of isotopically pure and Ge-doped Si epitaxial layers from 300 to 550 K. *Phys. Rev. B* **70**, 235322 (2004).
8. Liu, J., Choi, G.-M. & Cahill, D.G. Measurement of the anisotropic thermal conductivity of molybdenum disulfide by the time-resolved magneto-optic Kerr effect. *Journal of Applied Physics* **116**, 233107 (2014).
9. Feser, J.P., Liu, J. & Cahill, D.G. Pump-probe measurements of the thermal conductivity tensor for materials lacking in-plane symmetry. *Review of Scientific Instruments* **85**, 104903 (2014).
10. Li, D., Zhao, P., Zhao, J.-C. & Cahill, D.G. Generation and detection of gigahertz surface acoustic waves using an elastomeric phase-shift mask. *J. Appl. Phys.* **114**, 143102 (2013).
11. Zhang, X. et al. Surface Brillouin scattering study of the surface excitations in amorphous silicon layers produced by ion bombardment. *Physical Review B* **58**, 13677-13685 (1998).
12. Gladden, J.R., So, J.H., Maynard, J.D., Saxe, P.W. & Le Page, Y. Reconciliation of ab initio theory and experimental elastic properties of Al2O3. *Applied Physics Letters* **85**, 392-394 (2004).
13. Volkova, E.I., Jones, I.A., Brooks, R., Zhu, Y. & Bichoutskaia, E. Sequential multiscale modelling of SiC/Al nanocomposites reinforced with WS2 nanoparticles under static loading. *Physical Review B* **86**, 104111 (2012).
14. Huang, F. & Weaver, M.L. Effective biaxial modulus of ideally (hkl)-fiber-textured hexagonal, tetragonal, and orthorhombic films. *Journal of Applied Physics* **100**, 093523 (2006).
15. O'Hara, K.E., Hu, X. & Cahill, D.G. Characterization of nanostructured metal films by picosecond acoustics and interferometry. *Journal of Applied Physics* **90**, 4852-4858 (2001).
16. Beal, A.R. & Hughes, H.P. Kramers-Kronig analysis of the reflectivity spectra of 2H-MoS 2 , 2H-MoSe 2 and 2H-MoTe 2. *Journal of Physics C: Solid State Physics* **12**, 881 (1979).
